# Redetermined crystal structure of β-dl-me­thio­nine at 320 K

**DOI:** 10.1107/S2056989015008749

**Published:** 2015-05-13

**Authors:** Carl Henrik Görbitz, Jan Christian Paulsen, Jon Borgersen

**Affiliations:** a Department of Chemistry, University of Oslo, PO Box 1033 Blindern, N-0315 Oslo, Norway; b Department of Physics, University of Oslo, PO Box 1048 Blindern, N-0316 Oslo, Norway

**Keywords:** crystal structure, amino acid, phase transition, disorder

## Abstract

The structure of β-dl-me­thio­nine, C_5_H_11_NO_2_S, in the space group *C*2/*c*, is here confirmed to be fully ordered all the way up to the phase transition at approximately 326 K, where displacive sliding of mol­ecular bilayers gives the disordered *P*2_1_/*c* α form [data at 340 K; Görbitz (2014). *Acta Cryst.* E**70**, 341–343]. The geometry of hydrogen bonds in LD–LD hydrogen-bonding patterns [Görbitz *et al.* (2009). *Acta Cryst.* B**65**, 393–400] at the hydro­philic core of each mol­ecular bilayer are virtually unperturbed by the phase shift, but the C—C—S—C torsion angle of the side chain changes from *trans* at 320 K to *gauche*+ for the major conformation at 340 K.

## Related literature   

For previous investigations of dl-me­thio­nine (dl-Met), see: Mathieson (1952 ▸); Taniguchi *et al.* (1980 ▸); Alagar *et al.* (2005 ▸); Görbitz (2014 ▸); Görbitz *et al.* (2014 ▸). For a discussion of displacive phase transitions of amino acids with linear side chains and structures of quasiracemic complexes, see: Görbitz & Karen (2015 ▸). For the phase behaviour of the corresponding enanti­omeric substances, including l-Met and l-norvaline, see: Görbitz *et al.* (2015 ▸). For a discussion of hydrogen-bonding patterns in the crystal structures of hydrophobic amino acids, see: Görbitz *et al.* (2009 ▸).
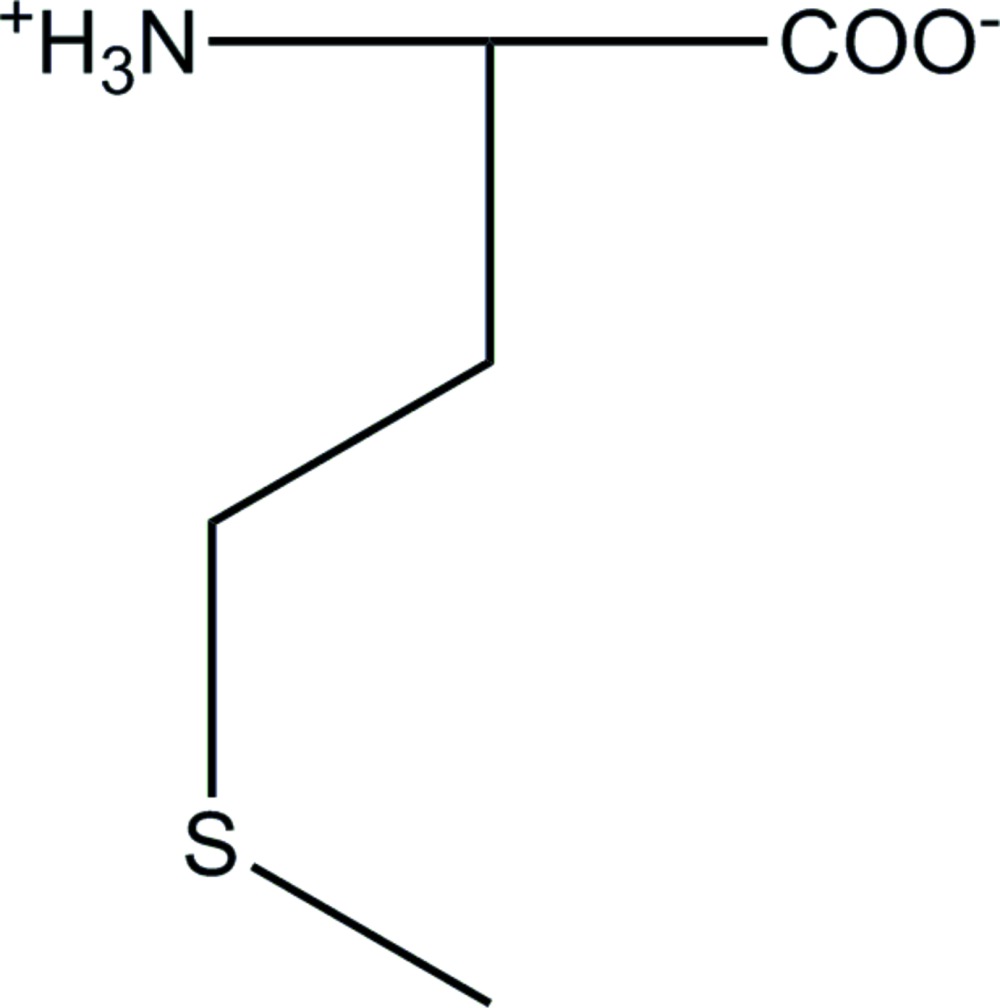


## Experimental   

### Crystal data  

C_5_H_11_NO_2_S
*M* *_r_* = 149.21
Monoclinic, 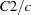

*a* = 31.774 (2) Å
*b* = 4.6969 (3) Å
*c* = 9.8939 (7) Å
β = 91.224 (2)°
*V* = 1476.20 (18) Å^3^
*Z* = 8
Mo *K*α radiation
μ = 0.37 mm^−1^
*T* = 320 K
0.72 × 0.15 × 0.10 mm

### Data collection  

Bruker D8 Advance single-crystal CCD diffractometer
Absorption correction: multi-scan (*SADABS*; Bruker, 2014 ▸) *T*_min_ = 0.924, *T*_max_ = 1.000
10516 measured reflections
2060 independent reflections
1567 reflections with *I* > 2σ(*I*)
*R*_int_ = 0.031

### Refinement  

*R*[*F* ^2^ > 2σ(*F* ^2^)] = 0.046
*wR*(*F* ^2^) = 0.116
*S* = 1.03
2060 reflections
92 parameters
H atoms treated by a mixture of independent and constrained refinement
Δρ_max_ = 0.34 e Å^−3^
Δρ_min_ = −0.39 e Å^−3^

### 

Data collection: *APEX2* (Bruker, 2014 ▸); cell refinement: *SAINT-Plus* (Bruker, 2014 ▸); data reduction: *SAINT-Plus*; program(s) used to solve structure: *SHELXS2014* (Sheldrick, 2008 ▸); program(s) used to refine structure: *SHELXL2014* (Sheldrick, 2015 ▸); molecular graphics: *Mercury* (Macrae *et al.*, 2008 ▸); software used to prepare material for publication: *SHELXL2014*.

## Supplementary Material

Crystal structure: contains datablock(s) I, global. DOI: 10.1107/S2056989015008749/su5129sup1.cif

Structure factors: contains datablock(s) I. DOI: 10.1107/S2056989015008749/su5129Isup2.hkl

**Fig. 1.**
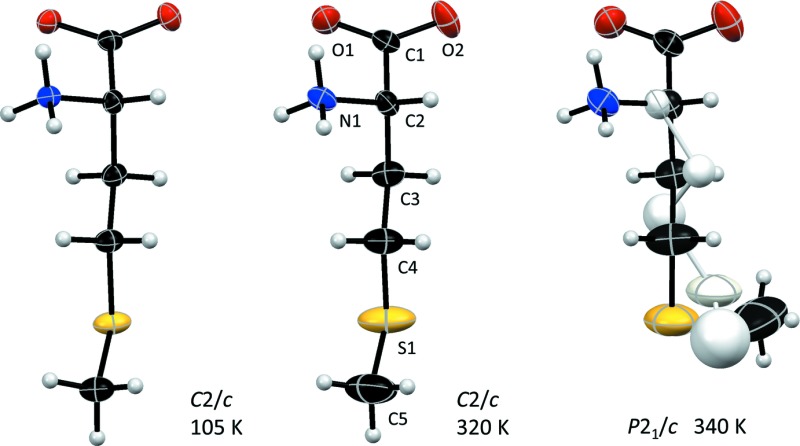
The molecular structure of β-DL-methionine at 320 K, with atom labelling, flanked by the structures at 105 K (published with the alternative space group setting I2/a, Alagar et al., 2005; Görbitz, 2014) and 340 K (Görbitz et al., 2014). Thermal displacement ellipsoids are shown at the 50% probability level. Atoms of the minor side-chain conformation with occupancy 0.0491 (18) at 340 K (with H atoms omitted) are shown in a lighter tone. The side-chain conformation is gauche–, trans, trans (as defined by the N1—C2—C3—C4, C2—C3—C4—S1 and C3—C4—S1—C5 torsion angles of the L-enantiomer shown) at 105 and 320 K, while the major and minor conformations at 340 K are gauche–, trans, gauche+ and gauche+, trans, gauche+, respectively.

Supporting information file. DOI: 10.1107/S2056989015008749/su5129Isup3.cml

dl I a et al. et al. gauche trans trans l gauche trans gauche+ gaucsu5129.cif he trans gauche+ . . DOI: 10.1107/S2056989015008749/su5129fig1.tif
The mol­ecular structure of β-dl-me­thio­nine at 320 K, with atom labelling, flanked by the structures at 105 K (published with the alternative space group setting *I*2/*a*, Alagar *et al.*, 2005; Görbitz, 2014) and 340 K (Görbitz *et al.*, 2014). Thermal displacement ellipsoids are shown at the 50% probability level. Atoms of the minor side-chain conformation with occupancy 0.0491 (18) at 340 K (with H atoms omitted) are shown in a lighter tone. The side-chain conformation is *gauche*–, *trans*, *trans* (as defined by the N1—C2—C3—C4, C2—C3—C4—S1 and C3—C4—S1—C5 torsion angles of the l-enanti­omer shown) at 105 and 320 K, while the major and minor conformations at 340 K are *gauche*–, *trans*, *gauche+* and *gaucsu5129.cif he*+, *trans*, *gauche+*, respectively*.*

CCDC reference: 1063335

Additional supporting information:  crystallographic information; 3D view; checkCIF report

## Figures and Tables

**Table 1:** Hydrogen-bond geometry (, )

*D*H*A*	*D*H	H*A*	*D* *A*	*D*H*A*
N1H1O1^i^	0.90(2)	1.88(2)	2.7732(17)	173.7(19)
N1H2O2^ii^	0.92(2)	1.92(2)	2.8264(18)	171.0(18)
N1H3O2^iii^	0.90(2)	1.94(2)	2.7973(18)	159.2(18)

Symmetry codes: (i) 
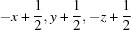
; (ii) 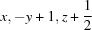; (iii) 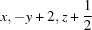.

**Table 2:** Selected torsion angles ()

Torsion angle	-DL-Met, 320K	-DL-Met, 340K*^*a*^*	-DL-Met, 340K*^*b*^*
N1C2C3C4	55.52(18)	59.3(4)	73(8)
C1C2C3C4	175.03 (14)	178.0(2)	78(5)
C2C3C4S1	179.16(12)	176.7(2)	178(5)
C3C4S1C5	174.55(16)	69.4(3)	60(3)

Notes: (*a*) major conformation, occupancy 0.9509(18) (Grbitz *et al.*, 2014 ▸); (*a*) minor conformation, occupancy 0.0491(18).

## supplementary crystallographic information

### S1. Comment

The two known polymorphs of DL-methionine (DL-Met), called α and β, were originally described by Mathieson (1952). Taniguchi *et al.* (1980) established the (first and only) transition temperature *T*_1_ to be about 326 K and carried out redeterminations at room temperature (β-form) and 333 K (α-form). *β*-DL-Met was subsequently redetermined at 105 K (Alagar *et al.*, 2005; Görbitz, 2014) and α-DL-Met at 340 K (Görbitz *et al.*, 2014). The transition between the two forms involves displacive sliding of molecular bilayers in the crystal. Recently, we have shown that such transitions are not limited to regular racemates of amino acids with linear side chains, but occur also for their quasiracemic complexes, including L-norvaline:D-norleucine (Görbitz & Karen, 2015), and for enantiomeric L-norvaline (Görbitz *et al.*, 2015). A special property of just these two structures is that all side chains remain ordered up to *T*_1_, but that one or more additional low-occupancy conformations appear at higher temperatures.

The side chain of β-DL-Met is ordered at 105 K (Alagar *et al.*, 2005; Görbitz, 2014), while a minor component is found for α-DL-Met at 340 K (Görbitz *et al.*, 2014), Fig. 1. As the quality of the available room-temperature crystal structure is rather low (*R* = 0.088; Taniguchi *et al.*, 1980), it was not known, however, if disorder in this case develops gradually between 105 K and 326 K (*T*_1_) or is introduced abruptly during the phase transition. The purpose of the present investigation was to settle this matter by collection of accurate experimental data with modern equipment at a temperature just below *T*_1._ This initiative was triggered by new results for L-Met (Görbitz *et al.*, 2015) that unexpectedly revealed disorder for both molecules in the asymmetric unit at room temperature.

The molecular structure of β-DL-Met at 320 K (I), depicted in Fig. 1, proves to be very well defined with no significant residual peaks in the electron density map. This means that the change from ordered to disordered side chain upon heating through a displacive phase transition, as observed for L-norvaline:D-norleucine and L-norvaline (see above), recurs for DL-Met. There is, however, one important tweak: while the dominating, major side-chain conformation of disordered molecules in the two other systems is always inherited from the ordered, lower-temperature polymorph, Fig. 1 shows that the *gauche*–, *trans*, *trans* conformation of β-DL-Met at 320 K (Table 1), is instead replaced by a *gauche*–, *trans*, *gauche*+ conformation in α-DL-Met at 340 *K*.

Hydrogen-bond parameters are listed in Table 2. N···O distances are marginally shorter at 320 K than at 340 K (Görbitz *et al.*, 2014).

### S2. Experimental

From a saturated solution of DL-Met in water (approximately 30 mg mL^-1^) 30 µL was pipetted into a 40 × 8 mm test tube, which was then sealed with parafilm. A needle was used to pierce a small hole in the parafilm and the tube placed inside a larger test tube filled with 2 ml of acetonitrile. The system was ultimately capped and left for one week at 20 °C. Suitable single crystals in the shape of needles and plates formed as the organic solvent diffused into the aqueous solution.

### S3. Refinement

Coordinates were refined for amino H atoms with *U*_iso_(H) = 1.5*U*_eq_(N). The C-bound H atoms were positioned with idealized geometry and treated as riding atoms: C—H = 0.96 - 0.98 Å with *U*_iso_(H) = 1.5*U*_eq_(C) for methyl H atoms, allowing free rotation for the terminal side-chain methyl group, and with 1.2*U*_eq_(C) for other H atoms.

### Crystal data

**Table:**

C_5_H_11_NO_2_S	*F*(000) = 640
*M**_r_* = 149.21	*D*_x_ = 1.343 Mg m^−^^3^
Monoclinic, *C*2/*c*	Mo *K*α radiation, λ = 0.71073 Å
*a* = 31.774 (2) Å	Cell parameters from 4136 reflections
*b* = 4.6969 (3) Å	θ = 2.6–29.5°
*c* = 9.8939 (7) Å	µ = 0.37 mm^−^^1^
β = 91.224 (2)°	*T* = 320 K
*V* = 1476.20 (18) Å^3^	Needle, colourless
*Z* = 8	0.72 × 0.15 × 0.10 mm

### Data collection

**Table:**

Bruker D8 Advance single-crystal CCD diffractometer	2060 independent reflections
Radiation source: fine-focus sealed tube	1567 reflections with *I* > 2σ(*I*)
Graphite monochromator	*R*_int_ = 0.031
Detector resolution: 8.3 pixels mm^-1^	θ_max_ = 29.5°, θ_min_ = 2.6°
Sets of exposures each taken over 0.5° ω rotation scans	*h* = −44→39
Absorption correction: multi-scan (*SADABS*; Bruker, 2014)	*k* = −6→6
*T*_min_ = 0.924, *T*_max_ = 1.000	*l* = −13→13
10516 measured reflections	

### Refinement

**Table:**

Refinement on *F*^2^	0 restraints
Least-squares matrix: full	Hydrogen site location: inferred from neighbouring sites
*R*[*F*^2^ > 2σ(*F*^2^)] = 0.046	H atoms treated by a mixture of independent and constrained refinement
*wR*(*F*^2^) = 0.116	*w* = 1/[σ^2^(*F*_o_^2^) + (0.0458*P*)^2^ + 1.6546*P*] where *P* = (*F*_o_^2^ + 2*F*_c_^2^)/3
*S* = 1.03	(Δ/σ)_max_ = 0.001
2060 reflections	Δρ_max_ = 0.34 e Å^−^^3^
92 parameters	Δρ_min_ = −0.39 e Å^−^^3^

### Special details

**Table:**

Geometry. All e.s.d.'s (except the e.s.d. in the dihedral angle between two l.s. planes) are estimated using the full covariance matrix. The cell e.s.d.'s are taken into account individually in the estimation of e.s.d.'s in distances, angles and torsion angles; correlations between e.s.d.'s in cell parameters are only used when they are defined by crystal symmetry. An approximate (isotropic) treatment of cell e.s.d.'s is used for estimating e.s.d.'s involving l.s. planes.
Refinement. Final *R*-factor is 0.0364 for a refinement based on 1300 reflections with 2 theta < 50 °.

### Fractional atomic coordinates and isotropic or equivalent isotropic displacement parameters (Å^2^)

**Table:**

	*x*	*y*	*z*	*U*_iso_*/*U*_eq_	
S1	0.44096 (2)	0.65785 (16)	0.45693 (7)	0.0684 (2)	
O1	0.28350 (4)	0.4246 (2)	0.19616 (11)	0.0316 (3)	
N1	0.29732 (4)	0.7975 (3)	0.39652 (12)	0.0252 (3)	
H1	0.2705 (6)	0.826 (4)	0.3685 (19)	0.038*	
H2	0.2998 (6)	0.627 (5)	0.441 (2)	0.038*	
H3	0.3042 (6)	0.926 (4)	0.461 (2)	0.038*	
C1	0.30472 (4)	0.6339 (3)	0.16326 (14)	0.0225 (3)	
C2	0.32477 (5)	0.8093 (3)	0.27735 (13)	0.0229 (3)	
H21	0.3278	1.0073	0.2479	0.028*	
O2	0.31323 (4)	0.7062 (3)	0.04532 (10)	0.0383 (3)	
C3	0.36823 (5)	0.6847 (4)	0.31133 (16)	0.0318 (3)	
H31	0.3651	0.4828	0.3293	0.038*	
H32	0.3858	0.7044	0.2329	0.038*	
C4	0.39054 (5)	0.8220 (4)	0.43157 (19)	0.0406 (4)	
H41	0.3739	0.7986	0.5118	0.049*	
H42	0.3940	1.0242	0.4153	0.049*	
C5	0.45831 (8)	0.8257 (7)	0.6094 (3)	0.0750 (8)	
H51	0.4861	0.7596	0.6334	0.113*	
H52	0.4393	0.7799	0.6804	0.113*	
H53	0.4589	1.0282	0.5965	0.113*	

### Atomic displacement parameters (Å^2^)

**Table:**

	*U*^11^	*U*^22^	*U*^33^	*U*^12^	*U*^13^	*U*^23^
S1	0.0405 (3)	0.0825 (5)	0.0809 (4)	0.0201 (3)	−0.0269 (3)	−0.0255 (4)
O1	0.0365 (6)	0.0287 (6)	0.0295 (6)	−0.0081 (5)	−0.0036 (4)	−0.0017 (4)
N1	0.0289 (6)	0.0278 (6)	0.0189 (6)	0.0044 (5)	−0.0031 (5)	−0.0035 (5)
C1	0.0270 (7)	0.0198 (6)	0.0204 (6)	0.0041 (5)	−0.0051 (5)	−0.0015 (5)
C2	0.0308 (7)	0.0192 (6)	0.0186 (6)	−0.0018 (5)	−0.0028 (5)	0.0008 (5)
O2	0.0657 (8)	0.0314 (6)	0.0176 (5)	−0.0024 (6)	−0.0001 (5)	0.0011 (4)
C3	0.0278 (7)	0.0356 (8)	0.0318 (8)	0.0003 (7)	−0.0033 (6)	−0.0050 (7)
C4	0.0336 (8)	0.0417 (10)	0.0458 (10)	0.0047 (7)	−0.0144 (7)	−0.0078 (8)
C5	0.0579 (14)	0.104 (2)	0.0620 (14)	0.0004 (14)	−0.0292 (12)	−0.0068 (15)

### Geometric parameters (Å, º)

**Table:**

S1—C5	1.779 (2)	C2—H21	0.9800
S1—C4	1.7907 (17)	C3—C4	1.516 (2)
O1—C1	1.2398 (18)	C3—H31	0.9700
N1—C2	1.4825 (18)	C3—H32	0.9700
N1—H1	0.90 (2)	C4—H41	0.9700
N1—H2	0.92 (2)	C4—H42	0.9700
N1—H3	0.90 (2)	C5—H51	0.9600
C1—O2	1.2504 (18)	C5—H52	0.9600
C1—C2	1.5258 (19)	C5—H53	0.9600
C2—C3	1.530 (2)		
			
C5—S1—C4	100.84 (11)	C4—C3—H31	108.6
C2—N1—H1	108.6 (12)	C2—C3—H31	108.6
C2—N1—H2	111.8 (12)	C4—C3—H32	108.6
H1—N1—H2	110.3 (17)	C2—C3—H32	108.6
C2—N1—H3	113.4 (12)	H31—C3—H32	107.6
H1—N1—H3	109.4 (17)	C3—C4—S1	109.19 (12)
H2—N1—H3	103.2 (16)	C3—C4—H41	109.8
O1—C1—O2	126.16 (13)	S1—C4—H41	109.8
O1—C1—C2	117.06 (12)	C3—C4—H42	109.8
O2—C1—C2	116.66 (13)	S1—C4—H42	109.8
N1—C2—C1	108.95 (12)	H41—C4—H42	108.3
N1—C2—C3	110.76 (11)	S1—C5—H51	109.5
C1—C2—C3	108.45 (12)	S1—C5—H52	109.5
N1—C2—H21	109.6	H51—C5—H52	109.5
C1—C2—H21	109.6	S1—C5—H53	109.5
C3—C2—H21	109.6	H51—C5—H53	109.5
C4—C3—C2	114.47 (13)	H52—C5—H53	109.5
			
O1—C1—C2—N1	−31.43 (17)	C2—C3—C4—S1	−179.16 (12)
O2—C1—C2—N1	152.41 (13)	C3—C4—S1—C5	−174.55 (16)
O1—C1—C2—C3	89.21 (16)	H1—N1—C2—C1	−44.4 (13)
O2—C1—C2—C3	−86.95 (16)	H2—N1—C2—C1	76.5 (13)
N1—C2—C3—C4	−55.52 (18)	H3—N1—C2—C1	−167.3 (14)
C1—C2—C3—C4	−175.03 (14)		

### Hydrogen-bond geometry (Å, º)

**Table:**

*D*—H···*A*	*D*—H	H···*A*	*D*···*A*	*D*—H···*A*
N1—H1···O1^i^	0.90 (2)	1.88 (2)	2.7732 (17)	173.7 (19)
N1—H2···O2^ii^	0.92 (2)	1.92 (2)	2.8264 (18)	171.0 (18)
N1—H3···O2^iii^	0.90 (2)	1.94 (2)	2.7973 (18)	159.2 (18)

Symmetry codes: (i) −*x*+1/2, *y*+1/2, −*z*+1/2; (ii) *x*, −*y*+1, *z*+1/2; (iii) *x*, −*y*+2, *z*+1/2.
