# Supplementary figures and images for: Redetermined crystal structure of β-dl-me­thio­nine at 320 K
Source: Acta Crystallogr E Crystallogr Commun. 2015 May 13;71(Pt 6):o398–9. doi: 10.1107/S2056989015008749 (PMC4459326; doi:10.1107/S2056989015008749)

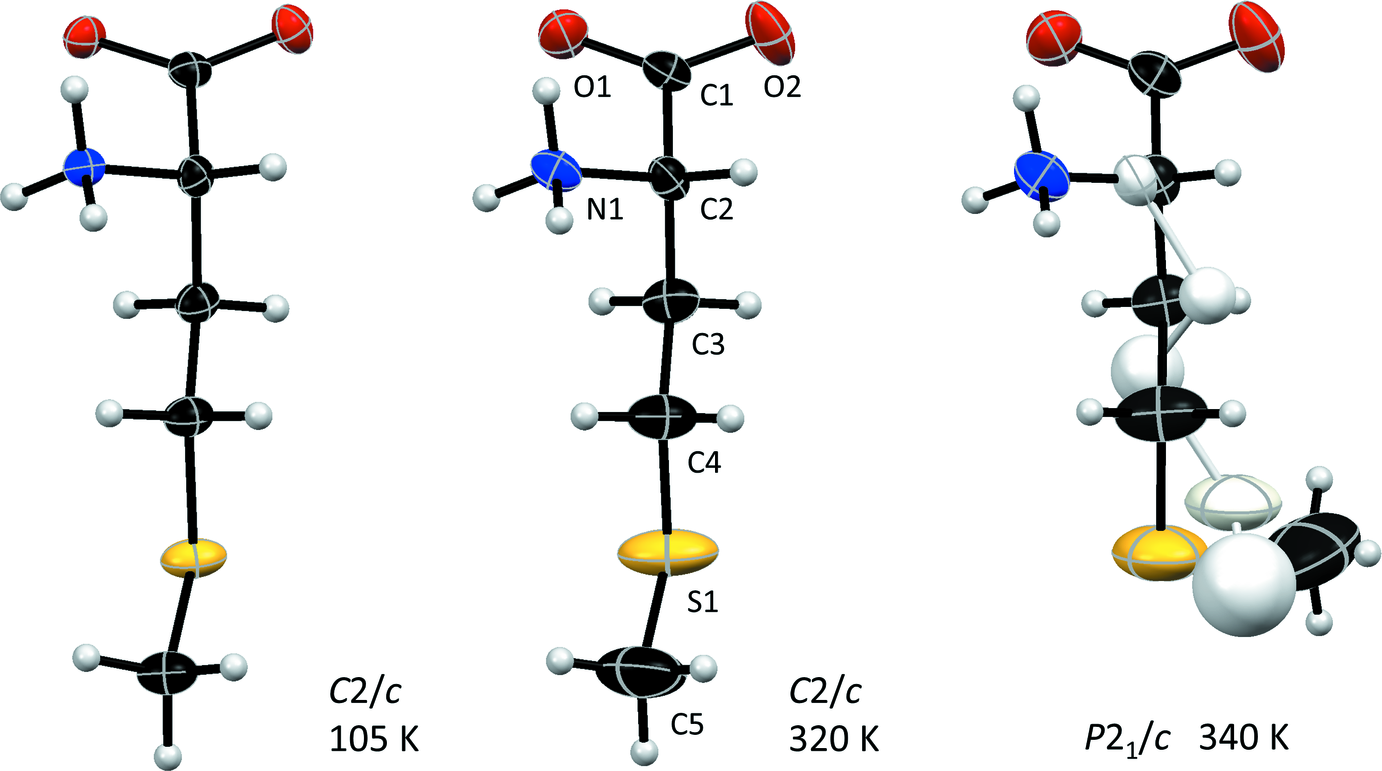

Supplement: Supplementary file 4 [file e-71-0o398-fig1.tif]
